# An improved 96-well turbidity assay for T4 lysozyme activity

**DOI:** 10.1016/j.mex.2015.05.004

**Published:** 2015-05-18

**Authors:** Tasha B. Toro, Thao P. Nguyen, Terry J. Watt

**Affiliations:** Department of Chemistry, Xavier University of Louisiana, New Orleans, LA 70125, United States

**Keywords:** 96-well turbidity assay for T4 lysozyme activity, T4 lysozyme, Quantitative, Turbidity assay, Metal affinity chromatography, Protein expression

## Abstract

T4 lysozyme (T4L) is an important model system for investigating the relationship between protein structure and function. Despite being extensively studied, a reliable, quantitative activity assay for T4L has not been developed. Here, we present an improved T4L turbidity assay as well as an affinity-based T4L expression and purification protocol. This assay is designed for 96-well format and utilizes conditions amenable for both T4L and other lysozymes. This protocol enables easy, efficient, and quantitative characterization of T4L variants and allows comparison between different lysozymes. Our method:

• Is applicable for all lysozymes, with enhanced sensitivity for T4 lysozyme compared to other 96-well plate turbidity assays;
• Utilizes standardized conditions for comparing T4 lysozyme variants and other lysozymes; and
• Incorporates a simplified expression and purification protocol for T4 lysozyme.

## Method

### T4 lysozyme expression

Genes encoding T4 lysozyme (T4L) and an enzymatically inactive variant, T4L-E11H, were cloned into an expression system which allows easy expression in *E. coli* and purification. A pseudo-wild-type cysteine-free T4L and T4L-E11H (also cysteine-free) were amplified from Addgene constructs 18111 and 18226, respectively, and cloned into pJExpress (DNA2.0) with a C-terminal TEV protease cleavage site and His_6_ tag.

1. Grow DH5α *E. coli* harboring the T4L-containing plasmid at 37 °C to OD_600_ 1.0.
2. Induce expression with 1 mM IPTG and incubate cells at 30 °C for an additional hour. Note that additional growth time following induction may result in lower yield due to lysis by T4 lysozyme.
3. Spin down cells at 3000 × *g* for 20 min at 4 °C. Proceed immediately to purification or store the pellet at −20 °C overnight, or −80 °C for longer periods.

### Metal affinity purification

We used a standard batch/column purification with TALON resin (Clontech), with the specific variations described below. However, any metal affinity purification should work, provided the sodium chloride concentration is kept high to improve protein solubility.

1. Lyse cells by incubation in lysis buffer [30 mM potassium phosphate pH 7.6, 0.5 M NaCl, 5% glycerol, 5 mM Imidazole pH 8.0, 2 mM MgCl_2_, 1X HALT protease inhibitor (Thermo Scientific), 0.5 mg/mL^−1^ hen egg white lysozyme (HEWL; MP Biomedicals)] followed by sonication for 3 × 10 s on ice.
2. Clarify resin at 27,000 × *g* for 20 min at 4 °C.
3. Incubate clarified lysate with TALON resin (Clontech) for 15 min at room temperature, according to the manufacturer’s instructions.
4. Wash resin twice with 10 bed volumes of wash buffer (30 mM potassium phosphate pH 7.6, 0.5 M NaCl, 5% glycerol, 5 mM Imidazole pH 8.0), according to the manufacturer’s instructions.
5. Transfer the resin to column housing, and wash once more with 10 bed volumes of wash buffer.
6. Elute the protein with elution buffer (30 mM potassium phosphate pH 7.6, 0.5 M NaCl, 5% glycerol, 150 mM Imidazole pH 8.0), collected in 1 bed volume fractions.
7. To cleave the His_6_ tag of T4 lysozyme, add His_6_-tagged tobacco etch virus (TEV) protease (1:25) to protein-containing fractions, and pool the protein-containing fractions for dialysis into TEV cleavage buffer (30 mM potassium phosphate pH 7.6, 0.5 M NaCl, 5% glycerol, 1 mM 2-mercaptoethanol, 0.5 mM EDTA) overnight at 4 °C with one buffer change.
8. Dialyze the protein into buffer containing 30 mM potassium phosphate pH 7.6, 200 mM NaCl, and 5% glycerol overnight at 4 °C with one buffer change.
9. Flow the protein over a TALON resin column equilibrated with dialysis buffer and collect the flow-through, to remove proteins which non-specifically bind the resin and to remove the TEV protease.
10. Store the purified protein at 4 °C in 30 mM potassium phosphate pH 7.6, 200 mM NaCl, 5% glycerol, and 1 mM tris (2-carboxyethyl) phosphine **(**TCEP).

**Fig. 1:**
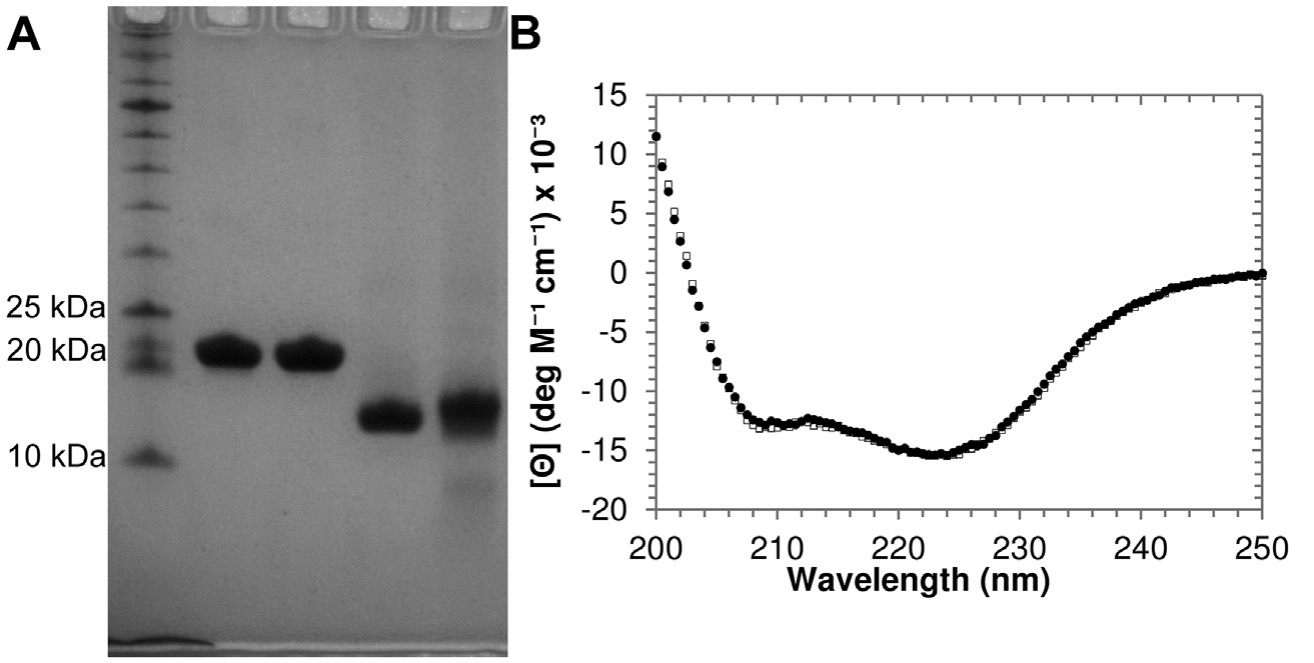
Confirmation of T4 lysozyme purity and native structure. (A) Approximately 5 μg of each T4 lysozyme variant assayed were subjected to analysis by SDS-PAGE, followed by protein staining with Gelcode Blue (Thermo Scientific). Lane 1–NEB protein ladder (10–250 kDa); lane 2—T4L; lane 3—T4L-E11H; lane 4—HEWL; lane 5—HL. Under these conditions, any contaminating proteins present at levels of 10 ng or greater (0.2%) would appear, and so our preparations of T4L and T4L-E11H are at least 99% pure. The commercially obtained HL appears slightly less pure. (B) Proteins purified during this study were monitored by CD spectrophotometry. The spectra of 1.0 μM wild-type (black circles) and E11H variant (open squares) in 30 mM potassium phosphate pH 7.2 are similar to each other and consistent with the expected structure. Spectra are an average of 4 accumulations obtained at a scan rate of 20 nm/min^−1^ with a 2 s integration every 0.5 nm in a 2 mm quartz cuvette.

This procedure typically yields 1–10 mg of highly purified protein (Fig. 1A) per liter of cell culture, and protein is stable for at least several months. Purified protein was monitored by circular dichroism spectrophotometry. The spectra of both wild-type T4 lysozyme and the E11H variant were consistent with properly folded protein under the reaction conditions, based on comparison to a previously reported CD spectrum for this protein [1] and the spectrum shape consistent with a primarily α-helical protein (Fig. 1B).

### Turbidity activity assay

We obtained human lysozyme (HL) (Sigma) and HEWL (MP Biomedicals) commercially as solids, although any source of lysozymes should be suitable for this assay. In general, we solubilized lysozyme at 1.0 mg/mL^−1^ in 30 mM potassium phosphate pH 7.6, 200 mM NaCl, 5% glycerol, 1 mM TCEP, to be consistent with the buffer of the purified T4 lysozyme.

1. Prepare a solution of 0.3 mg/mL^−1^ dried *Micrococcus luteus* (*M. lysodeikticus*; Sigma) in assay buffer (either 30 mM potassium phosphate pH 7.2 or 66 mM potassium phosphate pH 6.2) and vortex extensively (approximately 30–60 s) to suspend.
2. Allow the solution to settle for approximately 30 min before performing the assay.
3. Add lysozyme diluted to a final volume of 20 μL in assay buffer to wells of a flat-bottomed 96-well plate, in triplicate. We typically used 1.2 μg T4L and T4L-E11H, and 0.2 μg HL and HEWL.
4. Immediately before monitoring OD, add 200 μL of the cell suspension to each well of the 96-well plate containing lysozyme.
5. Allow reactions to proceed at 25 °C immediately after adding cell suspension (no additional mixing or shaking) and record the OD_450_ at 15 s intervals for 5 min. We utilized a Bio-Tek H1m plate reader.
6. Following data collection, calculate the average OD of triplicate wells at each timepoint for the set of technical replicates.
7. Obtain the slope using the average OD at each timepoint vs. time (minutes) for the consistently linear portion of the experiment. Under our assay conditions, the linear portion was generally the period 0–4 min for T4L and T4L-E11H, and 0–2 min for HEWL and HL.
8. Convert slopes to units, where 1 unit is equivalent to a decrease in absorbance of 0.001/min^−1^ [2], [3], [4], [5].
9. Subtract the units for a given lysozyme from the units for the corresponding buffer control and adjust according to the amount of enzyme present in the reaction, resulting in a measure of activity in Units μg^−1^.
10. To improve the reliability of the activity values, perform the experiment two additional times using independent substrate preparations. Calculate final activity values and standard deviations for the average of each experiment for each experimental condition.

## Methods validation

Using the assay described above, we compared the activity of T4L, T4L-E11H (previously reported as catalytically inactive; [6], [7]), HL, and HEWL using two different assay buffer conditions (Table 1). The first condition (30 mM potassium phosphate pH 7.2) was based on previous reports that T4L prefers a buffer with low ionic strength and a neutral pH [8]. The second condition (66 mM potassium phosphate pH 6.2) was previously reported for assessing activity of other lysozymes in similar assays [4], [9]. HL and HEWL were equivalently active in both buffer conditions; however, T4L only showed measurable activity in 30 mM potassium phosphate pH 7.2 (Table 1). These findings, as well as those of a sampling of additional ionic strength and pH conditions (data not shown), are in agreement with previous data suggesting that the optimal conditions of T4L are different and more restrictive than for HEWL [8]. An assay buffer of 30 mM potassium phosphate pH 7.2 allows for characterization of T4L activity as well as other lysozymes under common conditions, in contrast to the previously reported 66 mM potassium phosphate pH 6.2.

**Fig. 2:**
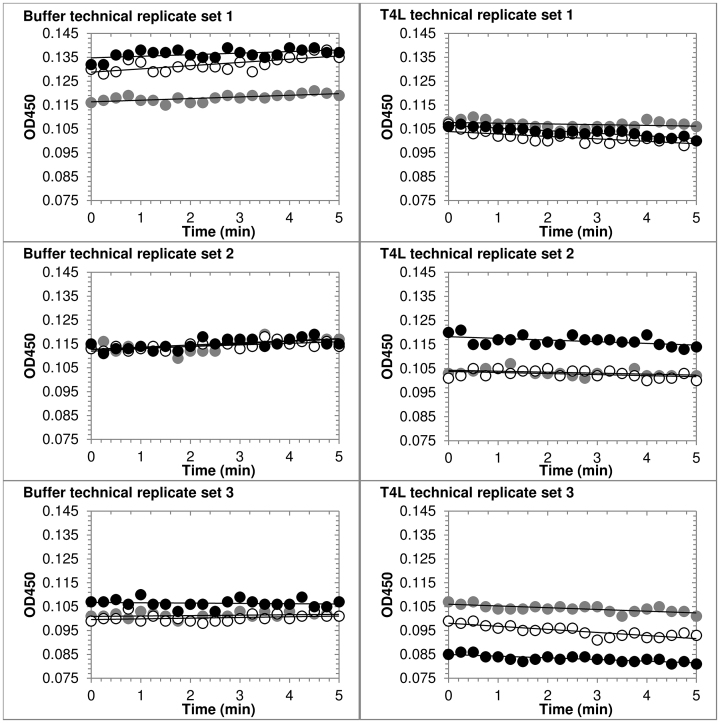
Activity of T4 lysozyme in low ionic strength buffer. *M. luteus* (0.3 mg/mL^−1^) in 30 mM potassium phosphate pH 7.2 was added to a 96-well plate in triplicate containing T4L (right) or buffer (left). OD_450_ was measured at 15 s intervals. Data shown represents technical triplicates for three independent experiments (each replicate series as white, grey, or black). Lines represent the best fit to each data set, to illustrate overall slope of the data within each replicate.

Although this protocol has a relatively low signal-to-noise ratio, the resulting slopes for the technical replicates within a single trial are consistent over an initial period of a few minutes, even when the absolute absorbance values vary substantially (Fig. 2). The reasonable agreement between triplicate experiments, represented by the standard deviations calculated from three independent trials (where each trial had 3 technical replicates), indicates that the activity measurement is quite reproducible (Table 1) despite using approximately 10-fold less lysozyme (all types) per reaction than has been reported in other methods [2], [9], [10], [11], [12], and with sensitivity similar to that of much more labor-intensive *E. coli*-based assays [13]. Thus, even though the signal-to-noise ratio is relatively low for a single replicate with this assay, performing technical triplicates within each of three independent experiments allows for the reliable quantification of lysozyme activity with significantly lower enzyme concentrations, and the signal intensity can be enhanced by using greater amounts of enzyme at the expense of a shorter linear reaction time.

Several parameters of the assay are critical for obtaining reproducible activity measurements for T4L. First, the 30 minute time allowance between cell suspension and the start of the assay was optimal for cells to rehydrate and settle appropriately. Allowing significantly less time for cell hydration resulted in more experimental noise. Conversely, allowing cells to settle for additional time resulted in a time-dependent decrease in the OD_450_ at the start of the experiment, also leading to a lower signal-to-noise ratio (data not shown). Furthermore, additional mixing performed by the plate reader, as reported in a similar lysozyme assay [2], resulted in increased noise without significantly affecting the activity measurements when performed during this protocol (data not shown). Finally, a highly purified enzyme is critical for generating reproducible activity values. Although the yield of T4L by our method is lower than the reported 16–23 mg L^−1^ yield for a previously developed and commonly used protocol [14], the exceptional purity of enzyme purified here, which itself likely contributes to the lower apparent protein yield, is more advantageous for this assay as well as other downstream applications. In addition, the purity obtained by sequential affinity chromatography is typically higher than that expected from another rapid purification method involving cation exchange [15].

When performing the protocol as described above, either round-bottom or flat-bottom 96-well plates can be used. In a less-effective variation of this protocol, cells were added to wells first and allowed to settle for 30 min in the assay plate. Lysozyme was then added to the well at the start of the assay. This method results in less noise for each well, but more variability between replicates (Table 2). In addition, this approach only worked with flat-bottom plates, as allowing the cells to settle in round-bottom plates resulted in extreme experimental noise.

## Additional information

T4L is an enzyme found in T4 phage that is tolerant of mutations and amenable to crystallization. Extensive mutagenesis and structural studies of this protein have been instrumental in our general understanding of the relationship between protein structure and function [16], [17]. While this enzyme has many advantages as a model system, a simple and robust activity assay has been lacking. The native function of lysozymes is to hydrolyze bacterial cells walls, and most assays exploit this activity. Several turbidity assays have been reported, which monitor the decrease in optical density of T4L-treated bacteria in suspension over time [8], [10], [12], [13], [18], [19]. Overall, these assays are cumbersome, sometimes involving extensive preparation of the cell wall substrates and utilizing cuvettes which can monitor only a single reaction at a time [10], [12], [13], [18], [19]. It has also been reported that these assays are only capable of generating internally normalized rates and not absolute activity [14]. Alternative assays have been developed for monitoring T4L activity; however, these assays also have significant drawbacks. For example, the halo assay requires a long incubation period and is not quantitative [20]. Others require specialized equipment, such as a CD spectrophotometer or HPLC while remaining low-throughput [1], [6], [21].

Multiple groups have reported activity assays for related lysozyme proteins, including HEWL and HL. These assays monitor the decrease in turbidity of suspended bacterial cells; however, the conditions and protocols reported for each of the assays are highly variable [2], [4], [9], [11], [22], [23], [24]. They are more robust than the assays reported for T4L, and some have even been adapted to 96-well format, but the assays often still suffer from substantial noise. Moreover, the assays do not work well for T4L, likely due in part to the relatively low activity level of T4L compared to the others and the high sensitivity of T4L to buffer conditions [8].

T4L-E11H was included in this study as a negative control, as the E11 residue was previously reported to be required for enzymatic activity [6], [7]. Based on these reports, we expected the E11H variant (as well as a buffer control) to result in no change in OD_450_ over time in our assay. Instead, the OD_450_ consistently increased during the course of the experiment when cells were incubated with T4L-E11H (Table 1). While we also observed this phenomenon with the buffer control, it was much more pronounced in the presence of T4L-E11H. This observation suggests that some of the T4L activity may be masked by the tendency of T4L to increase the OD_450_ in the absence of enzymatic activity, and that subtraction of T4L-E11H activity, rather than buffer, may be more appropriate for determining T4L activity. Nevertheless, we observed a decrease in turbidity only when active enzyme was present in the reaction, consistent with prior reports that decreased turbidity in this assay correlates with enzyme activity [8], [10], [12], [13], [18], [19]. Although the reason for the increase in OD_450_ is not known, it indicates that the activity measurements presented for T4L, both here and in other reports, may be lower than the actual activity of the enzyme. The mechanism behind the increase in light scattering revealed by our assay could be the subject of future studies using other catalytically inactive lysozymes.

Here, we have presented a simple, robust activity assay in 96-well format that can quantitatively measure the activity of T4L, and is also appropriate for evaluating other lysozyme variants. This assay is facile, rapid, cost-effective, and provides quantitative activity measurements for T4L as well as other lysozyme proteins using a single set of reaction conditions. By performing the assay in 96-well format, multiple lysozyme variants can be simultaneously assayed for activity, which will facilitate the characterization of lysozyme variants, such as in comparative studies of T4L examining the effects of mutations on activity and for comparisons between T4L and other lysozymes, and lead to a more comprehensive understanding of the relationship between protein structure and function.

## Figures and Tables

**Table 1:** Activity and standard deviation for each lysozyme variant under our fully optimized conditions (30 mM potassium phosphate pH 7.2) and using the optimized method with previously published buffer conditions (66 mM potassium phosphate pH 6.2).

	Activity U μg^−1^	
Lysozyme	30 mM phosphate pH 7.2	66 mM phosphate pH 6.2
T4L	1.1 ± 0.2	−0.2 ± 0.3
T4L-E11H	−1.9 ± 0.6	−0.6 ± 1.3
HEWL	25 ± 2	29 ± 2
HL	51 ± 5	38 ± 9

**Table 2:** Comparison of variability for turbidity assay protocols based on three independent experiments using each method.

	% Standard deviation	
Lysozyme	Method A^a^	Method B^b^
T4L	20	81
T4L-E11H	30	115
HEWL	8.6	64
HL	9.3	24

a Cells were allowed to settle in a centrifuge tube before addition to the 96-well plate containing enzyme (Figure 1; Table 1).

b Cells were allowed to settle in the 96-well plate, and enzyme was added to cells.
